# Changes in Expression and Cellular Localization of Rat Skeletal Muscle ClC-1 Chloride Channel in Relation to Age, Myofiber Phenotype and PKC Modulation

**DOI:** 10.3389/fphar.2020.00714

**Published:** 2020-05-15

**Authors:** Elena Conte, Adriano Fonzino, Antonio Cibelli, Vito De Benedictis, Paola Imbrici, Grazia Paola Nicchia, Sabata Pierno, Giulia Maria Camerino

**Affiliations:** ^1^Department of Pharmacy-Drug Sciences, University of Bari “Aldo Moro”, Bari, Italy; ^2^Department of Biosciences, Biotechnologies and Biopharmaceutics, University of Bari “Aldo Moro”, Bari, Italy

**Keywords:** chloride ion channel 1, ClC-1, protein kinase C (PCK), muscle development, muscle aging, subcellular localization

## Abstract

The ClC-1 chloride channel 1 is important for muscle function as it stabilizes resting membrane potential and helps to repolarize the membrane after action potentials. We investigated the contribution of ClC-1 to adaptation of skeletal muscles to needs induced by the different stages of life. We analyzed the ClC-1 gene and protein expression as well as mRNA levels of protein kinase C (PKC) alpha and theta involved in ClC-1 modulation, in soleus (SOL) and extensor digitorum longus (EDL) muscles of rats in all stage of life. The cellular localization of ClC-1 in relation to age was also investigated. Our data show that during muscle development ClC-1 expression differs according to phenotype. In fast-twitch EDL muscles ClC-1 expression increased 10-fold starting at 7 days up to 8 months of life. Conversely, in slow-twitch SOL muscles ClC-1 expression remained constant until 33 days of life and subsequently increased fivefold to reach the adult value. Aging induced a downregulation of gene and protein ClC-1 expression in both muscle types analyzed. The mRNA of PKC-theta revealed the same trend as ClC-1 except in old age, whereas the mRNA of PKC-alpha increased only after 2 months of age. Also, we found that the ClC-1 is localized in both membrane and cytoplasm, in fibers of 12-day-old rats, becoming perfectly localized on the membrane in 2-month-old rats. This study could represent a point of comparison helpful for the identification of accurate pharmacological strategies for all the pathological situations in which ClC-1 protein is altered.

## Introduction

The biophysical properties, structure, and role of the chloride channel (ClC-1) in muscle physiology have been extensively investigated for more than 20 years (Aromataris and Rychkov, 2006; Tang and Chen, 2011; Imbrici et al., 2015; Pedersen et al., 2016; Bækgaard Nielsen et al., 2017; Jentsch and Pusch, 2018; Park and MacKinnon, 2018; Wang et al., 2019). ClC-1, encoded by the *Clcn1* gene, is exclusively present in skeletal muscle (Steinmeyer et al., 1991; Pedersen et al., 2016) where it mediates the bulk of plasma membrane Cl conductance (gCl). ClC-1 is important for muscle function as it stabilizes resting membrane potential and helps to repolarize the membrane after action potentials (Bækgaard Nielsen et al., 2017; Jentsch and Pusch, 2018; Phillips and Trivedi, 2018; Ravenscroft et al., 2018). Today, it is known that different types of *Clcn1* mutations are responsible for dominant and recessive myotonia (Desaphy et al., 2013; Poroca et al., 2017). *Clcn1* mutations can induce a large number of functionality defects including not only the alteration of the biophysical behavior of the channel but also the modification of surface expression of the channel or the alteration of membrane trafficking (Imbrici et al., 2015). Considering the key role of ClC-1 channel in setting sarcolemmal electrical properties and consequently contractile response, its correlation with muscular phenotype is straightforward. Indeed fast-twitch muscles as EDL are characterized by a higher gCl and ClC-1 mRNA with respect to the slow-twitch SOL muscle (Pierno et al., 2002). In accordance with the greater presence of ClC-1 channels in fast-twitch muscles compared to slow-twitch muscles, recent evidence demonstrates that ClC-1 protein expression is higher in type IIa (fast-oxidative) fibers compared to type I (slow-oxidative) fibers (Thomassen et al., 2018). The importance of ClC-1 in determining muscle phenotype is also evident during muscle disuse, due to bed rest or microgravity, when phenotype myofiber transition from slow to fast was observed in parallel with the early modification of gCl and ClC-1 channel expression (Pierno et al., 2002). ClC-1 function is regulated by phosphorylation events, in particular by protein kinase C (PKC) (Rosenbohm et al., 1995; Rosenbohm et al., 1999). A change in the modulation of gCl by PKC has been demonstrated in numerous conditions of skeletal muscles such as aging (De Luca et al., 1994) and prolonged disuse (Pierno et al., 2007), suggesting that altered biochemical modulation can at least, in part, account for the change in gCl observed in these conditions. Little is known about the specific isoforms of PKC involved in ClC-1 modulation; some studies suggest that PKC-theta and PKC-alpha are the most important in ClC-1 functional regulation (Pierno et al., 2007; Camerino et al., 2014).

The exact cellular localization of the ClC-1 channel between the sarcolemma and t-tubules has been the subject of intense debate. The majority of studies suggest that ClC-1 is localized in t-tubules, while others present solid evidence of the channel localization at the sarcolemma (Lueck et al., 2010; Fahlke, 2011; DiFranco et al., 2011; Lamb et al., 2011). This controversy could be resolved assuming that different conditions, can promote ClC-1 translocation from the sarcolemma to the t-tubules, or vice versa (Papponen et al., 2005). Our hypothesis suggests that a fraction of the ClC-1 protein is localized in an intracellular pool and transported on the plasma membrane through different cell signaling pathways, such as the phosphorylation–dephosphorylation pathway (Papponen et al., 2005), as occurs for other transporters (e.g., GLUT4).

In line with the development of electrical properties of skeletal muscles during growth, gCl and ClC-1 expression change with age. In particular, the electrophysiological measure of gCl in native fibers shows that gCl increases with growth. In the EDL muscles of rats between 8-12 days of age gCl quickly increases; after 12 days gCl slowly increases up to adult age (Conte Camerino et al., 1989). In accordance with the increase of gCl, *Clcn1* gene expression also increases (Steinmeyer et al., 1991). No data are available on gCl in muscles before 7–8 days of life and during muscular development in SOL muscles. Previous studies in fast-twitch EDL muscles have demonstrated that the aging process in rat skeletal muscle is associated with a reduction of resting gCl and ClC-1 mRNA expression (Pierno et al., 1999).

The role of ClC-1 has been studied by analysis of correlation between gene expression of the channel and gCl; however, until now the correlation with the protein expression of ClC-1 is lacking, also due to the limitations, until recently, of efficient antibodies. Only some information is available about the transcriptional and posttranscriptional regulation of ClC-1 during the different stages of life. Even less is known about the subcellular localization of ClC-1 during muscle development and aging.

The present study aimed to investigate the role of ClC-1 in slow- and fast-twitch skeletal muscles during all stages of rat life through a systematic correlation analysis between trascriptional and posttranscriptional regulation. With the aim to gain more information about the role of different isoforms of PKC on the posttranscriptional modulation of ClC-1 during lifespan, we analyzed also the correlation between ClC-1 expression and the mRNA coding for PKC-alpha and PKC-theta isoforms. Assuming that during muscle development different cellular stimuli may induce a modification of subcellular localization of ClC-1, we have, also, characterized the cellular localization of ClC-1 in slow and fast muscles during development and aging.

## Materials and Methods

### Animals

All experiments were performed in accordance with the Italian Guidelines for the Use and Care of Laboratory Animals (Leg. Decree 2014, no. 26), which comply with the European Communities Council Directive of 24 November, 1986 (86/609/EEC) and Guidelines from Directive 2010/63/EU of the European Parliament on the protection of animals used for scientific purposes, and were approved by the General Direction of animal health care and veterinary drugs of the Italian Ministry of Health (Leg. Decree 26/2014; protocol authorization number: 171/2017 PR). All animal studies comply with the ARRIVE guidelines.

The age-stages selected for the study, based on the guidelines of Sengupta (2013), and number of animals are reported in **Table 1**. Wistar rats were purchased from Charles River Laboratories (Italy). The female rat with its pups was provided by Charles River Laboratories to obtain the P1, P7, and P12 animals which were sacrificed at the desired age. For the analysis of P33 to M27 animals male rats were used, while for P1 to P12 animals the gender was not determined. For the age-stages P1 and P7, due to the reduced size of the samples three muscles (SOL or EDL) obtained from two animals of the same litter were grouped together and considered as a single sample. For all other age-stages (P12 to M27) only muscles of individual limbs were used for the analysis.

**Table 1:** Experimental design.

Age-stages	Days or months of life	Number of rats	Abbreviation
Birth	1 Day	14	P1
Neonatal	7 Days	14	P7
Postnatal-weaning	12 Days	6	P12
Early-adolescent	33 Days	5	P33
Late-adolescent	47 Days	5	P47
Young-adult	2 Months	6	M2
Adult	8 Months	6	M8
Old	27 Months	6	M27

The first column reports the age-stages of rats used in the study; the second column reports the age of animals (expressed in days or months) at the date of the sacrifice, and the third column the number of animals used for each age-stage analyzed. The fourth column indicates the abbreviations used for each age-stage.

The animals were housed in a temperature-controlled (22°C) room with a 12:12-h light-dark cycle. Before EDL and SOL muscle dissection, the animals were deeply anesthetized with ketamine (100 mg/kg ip) and xylazine (10 mg/kg ip) and sacrificed with an overdose of anesthetic followed by cervical dislocation. The muscles were immediately frozen in liquid nitrogen or liquid nitrogen-isopentane and stored at −80°C for further analysis of gene and protein expression and immunohistochemistry.

### mRNA Expression Analysis of EDL and SOL Muscles by Quantitative Real-Time PCR

The qPCR experiments were conducted as previously described (Camerino et al., 2015). Briefly, 400 ng of total RNA was reverse transcribed using SuperScript II Reverse Transcriptase protocol (Life Technologies C.N. 18064-014). Real-time PCR was performed in triplicate using the Applied Biosystems Real-time PCR 7500 Fast system (United States). The mRNA expression of the genes was normalized to the best housekeeping gene beta-actin (*Actb*) selected from glyceraldehyde-3-phosphate dehydrogenase (*Gapdh*), phosphoribosyltransferase 1 (*Hprt1*) using BestKeeper and NorFinder software. TaqMan hydrolysis primer and probe gene expression assays were obtained from Life Technologies with the following assay IDs: *Clcn1* ID: Rn00565736_m1; *Pkct* ID: Rn01765050_m1; *Pkca* ID: Rn01496145_m1; *Actb* ID: Rn00667869_m1; *Hprt1* ID: Rn01527840_m1; and; and *Gapdh* ID: Rn_01775763_g1. All gene expression experiments were conducted following the MIQE guidelines (Bustin et al., 2009).

### Protein Expression Analysis of EDL and SOL Muscles by Western Blot

The EDL and SOL muscles of rats at different life-stages were used to analyze ClC-1 protein expression that was assessed as described by Camerino et al. (2019). Briefly, samples were homogenized in ice cold buffer containing 20 mM Hepes (pH 7.4), 2 mM EDTA, 0.2 mM EGTA, 0.3 M sucrose, 0.2 mM phenylmethylsulfonyl fluoride and protease inhibitors. Homogenates were centrifuged at 7,000*g* for 5 min at 4°C. The supernatant obtained was centrifuged at 50,000*g* for 1 h at 4°C, and the pellet was solubilized in 20–30 ml of the same buffer. Incubation in primary rabbit anti-ClC1 antibody (MyBiosource, cod: MBS714620, diluted 1:200) was carried out overnight at 4°C, and 1-h incubation in horseradish peroxidase conjugated secondary goat anti-rabbit IgM/IgG antibody (Sigma-Aldrich, 1:5,000) was carried out at room temperature. The bands were visualized with a chemiluminescent substrate (Clarity Western ECL Substrate, Bio-Rad) and signals recorded with a Chemidoc imaging system (Bio-Rad). For each sample, the relative intensity was calculated by normalizing the intensity of the β–actin protein band (diluted at 1:300, rabbit Anti-Actin, Santa Cruz Biotechnology) as reference standard. Validation experiments were performed to acquire additional information on ClC-1 antibody specificity (**Supplementary Material**).

### Histological and Double-Immunofluorescence Analyses of EDL and SOL Muscles

The EDL and SOL muscles of rats at different age-stages, covered with tissue-tek O.C.T. (Bio-Optica), were frozen in isopentane cooled in liquid nitrogen in a slightly stretched position and stored at −80°C. Serial cross sections (8-μm thick) were cut in a cryostat microtome set at −20°C (HM525 NX, Thermo Scientific). The double-immunofluorescence was peformed as described in previous studies (Nicchia et al., 2016). The antibodies against ClC-1 and beta-dystroglycan (β-DG) (Novocastra cod: NCL-b-DG) were diluted 1:200 and 1:500, respectively, in PBS-gelation. The antibodies against aquaporin-1 to test the noise background in immunofluorescence was (B-11) sc-25287 (Santa Cruz). Tissue sections were analyzed with a Leica DM RXA epifluorescence microscope and PL Fluotar 16X/0.5na objective (Leica, Heidelberg GmbH, Mannheim, Germany) as previously shown (Nicchia et al., 2016). For confocal image acquisition and analysis, a Leica TCS SP8 confocal microscope with a 20X and 100X HC PL Apo oil Cs2 objective and Leica LASX software were used (https://www.leica-microsystems.com).

### Pearson’s Correlation Coefficient Analysis

The Pearson’s correlation coefficient analysis (PCC) was used to study both the correlation of subcellular localization of ClC-1 and β-DG (used as a marker of membrane sarcolemma) in fluorescence and the correlation between the expression of two genes or gene and protein in relation to age. Colocalization of ClC-1 and β-DG from images was made as previously used by others to quantify protein colocalization from epifluorescence image Li et al., 2004; Babbey et al., 2006; Saitoh et al., 2009; Kanda et al., 2011), following with the guidelines (Comeau et al., 2006; Dunn et al., 2011; McDonald And Dunn, 2013). For image analysis, PCC coefficient values (Pcc-R) were determined using ImageJ2 software with the implementation of Fiji software. Pearson’s Pcc-R values were assessed on three images for each age-stage analyzed.

The statistical significance of correlation between two genes or gene and protein expression was calculated using the P value from Pearson’s coefficient analysis (P <0.05). The degree of correlation between two fluorescence signals, or two gene trends, or gene and protein expression trends was evaluated according to Pcc-R values, as previously described by Zinchuk and Wu (2013) and reported in **Table 2**.

**Table 2:** Interpretation of Pcc-R values.

Pcc-R	Degree of correlation
*0.1*	Very weak
*0.2*	Weak
*0.3*	More than weak
*0.4*	Less than moderate
*0.5*	Moderate
*0.6*	More than moderate
*0.7*	Less than strong
*0.8*	Strong
*0.9*	Very strong

Degree of correlation between two fuorescence signals, or two gene trends, or gene and protein expression trends for each coefficient value of Pcc-R are presented.

### Statistical Analysis

Statistical analyses were performed using one-way ANOVA followed by Fisher’s test to evaluate multiple comparisons between groups. P <0.05 was considered statistically significant. All data were expressed as mean +/− standard error of the mean (SEM). The statistical analyses and figure generation were performed using Prism 7.0 software.

## Results

### Time-Course Analysis of *Clcn1* Transcriptional Level in EDL and SOL Muscles in Rats From Birth to Old Age

**Figure 1:**
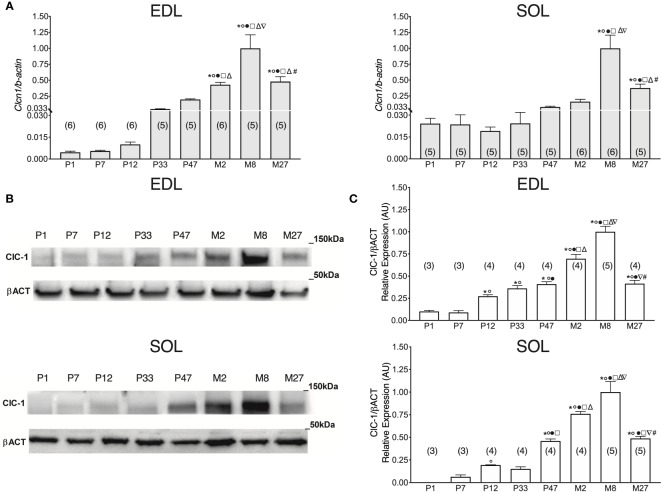
ClC-1 gene and protein expression analysis in EDL and SOL muscles of rats from birth to old age. **(A)** Histograms show the transcription level of *Clcn1* determined by qPCR and normalized with *Actb* in EDL and SOL muscles. **(B)** SDS-PAGE Western blot of ClC-1 and β-actin in EDL and SOL muscles at different ages. **(C)** Histograms show the densitometric analysis of ClC-1 bands expressed as a fraction of the respective β-actin band level in EDL and Sol muscles. Bars represent means ± SEM of the number of animals indicated in brackets. For graphical reasons, the values of single data were normalized on the mean of the M8 group. Significant differences between groups were assessed using one-way ANOVA (mRNA content of *Clcn1* in EDL muscles F=22.11, R=0.811; mRNA content of *Clcn1* in SOL muscles F=16.13, R=0.768; protein content of ClC-1 in EDL muscles F=58.78, R=0.947; protein content of ClC-1 in SOL muscles F=39.77, R=0.92). Fisher’s test was used to evaluate the individual differences between groups. Significant differences (P < 0.05) are expressed for * vs. P1, ° vs. P7, • vs. P12, □ vs. P33, Δ vs. P47, ∇ vs. M2, and # vs. M8.

To study the levels of *Clcn1* gene expression throughout all stages of life, we performed qPCR experiments in SOL and EDL muscles isolated from rats at different life-stages (**Figure 1A**). *Clcn1* mRNA was already detectable from birth in both SOL e EDL muscles. In agreement with previous studies (Lueck et al., 2007), *Clcn1* expression increased with muscle development but in a different manner in fast- and slow-twitch muscles. In EDL muscles *Clcn1* expression was constant during the neonatal age and thereafter gradually increased up to Month 8 (M8) (*Clcn1* gene expression increased 0.8-, 7.9-, 35-, 77-, and 181-fold in EDL muscles of P12, P33, P47, M2, and M8 rats, respectively, compared to P7 rats). In SOL muscles, *Clcn1* mRNA remained unaltered from P1 to M8. In accordance with previous studies (Pierno et al., 1999; Camerino et al., 2016), EDL muscles showed a significant reduction (~52%) of *Clcn1* gene expression in M27 rats compared to M8 rats. Our results also showed that in SOL muscles aging induces a reduction of ClC-1 mRNA (~62% in M27 rats compared to M8 rats).

It is well known that *Clcn1* gene expression differs in slow- and fast-twitch muscles. In particular, *Clcn1* mRNA was more expressed in fast-twitch muscles compared to slow-twitch muscles. However, it is unknown if this phenotype difference is present from birth or occurs during specific stages of muscle development. To obtain more information in this regard, we performed another set of experiments to observe the *Clcn1* expression in EDL and soleus muscles during development.

**Figure 2:**
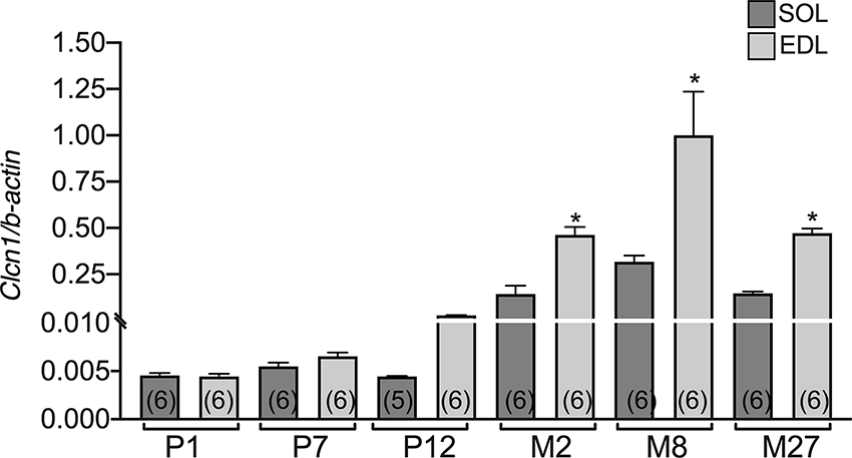
The difference of CLC-1 gene expression in EDL and SOL muscles from birth to old age. Histograms show the transcription level of *Clcn1* determined by qPCR and normalized with *Actb* in EDL and SOL muscles. Bars represent means ± SEM of the number of animals indicated in brackets. Significant differences between groups were evaluated using Student’s unpaired *t*-test. The significant difference was evaluated between EDL and SOL muscles in the same age-stage using Student’s unpaired *t*-test, with P <0.05 and expressed with *.

The difference between EDL and SOL muscles in *Clcn1* gene expression was not evident at birth and at the neonatal stage (P1 and P7) (**Figure 2**). A trend of greater *Clcn1* expression in EDL muscles compared to SOL muscles was observable at 12 days of life; this difference became significant in older rats. In particular, *Clcn1* gene expression was twofold greater in EDL compared to SOL muscles at all life-stages, starting from P12.

### Age-Related Expression of ClC-1 Protein in EDL and SOL Rat Muscles

Western blot analysis of the ClC-1 channel was performed to observe in parallel the corresponding level of ClC-1 protein from birth to old age in fast- and slow-twitch rat muscles.

In P1 rats, the ClC-1 protein was detectable only in EDL muscles and not in SOL muscles (**Figures 1B, C**). In EDL muscles, ClC-1 protein expression did not change between P1 and P7 rats, whereas in SOL muscles it was detectable only in P7 rats. ClC-1 protein expression significantly increased twofold in both SOL and EDL muscles in P12 rats compared to P7 rats. In EDL muscles, ClC-1 protein expression gradually increased from P12 rats to M8 rats (3.2-, 4.9-, 1.5-, and 2.6-fold in EDL muscles of P33, P47, M2, and M8 rats, respectively, compared to P12 rats). In SOL muscles, ClC-1 protein expression did not change between P12 and P33 rats, but significantly increased 2-, 4-, and 5.6-fold in P47, M2, and M8 rats, respectively, compared to P33 rats. The passage between the young-adult stages to adult stage (from M2 to M8) induced a significant increase of ClC-1 protein expression of 32% and 43% in both SOL and EDL muscles, respectively. In agreement with the *Clcn1* mRNA level, protein expression significantly decreased 58% and 51% in both the EDL and SOL muscles of old rats, respectively, compared to adult rats (from M8 to M27).

### Expression of *Pkct* and *Pkca* in EDL and SOL Muscles in Relation to Age

**Figure 3:**
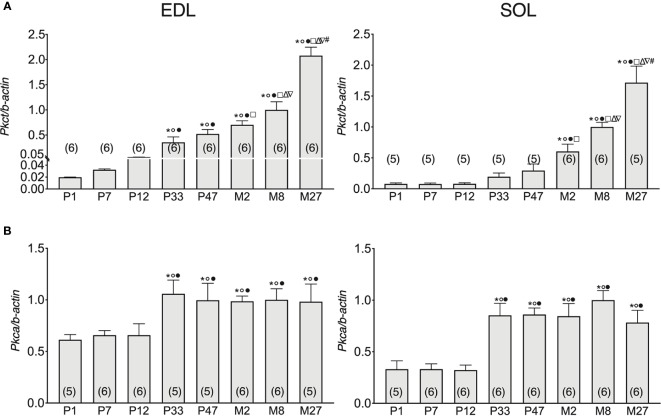
Pkca and Pkct gene expression analysis in EDL and SOL muscles of rats from birth to old age. Histograms show the transcription level of *Pkct* **(A)** and *Pkca* **(B)** performed by qPCR and normalized with *Actb*. Bars represent means ± SEM of the number of animals indicated in brackets. For graphical reasons, the values of the single data were normalized on the mean of the M8 group. Significant differences between groups were evaluated using one-way ANOVA (mRNA content of *Pkct* in EDL muscles F=48.35, R=0.89; mRNA content of *Pkct* in SOL muscles F=25.61, R=0.84; mRNA content of *Pkca* in EDL muscles F=2.96, R=0.36; mRNA content of *Pkca* in SOL muscles F=9.57, R=0.93). Fisher’s test was used to evaluate the individual differences between groups. Significant differences (P <0.05) are expressed for * vs. P1, ° vs. P7, • vs. P12, □ vs. P33, Δ vs. P47, ∇ vs. M2, and # vs. M8.

Previous studies have highlighted that ClC-1 phosphorylation by PKC induces the closure of the channel resulting in the reduction of gCl (Rosenbohm et al., 1995; Rosenbohm et al., 1999; Camerino et al., 2014; Pedersen et al., 2016), therefore, we set out to analyze the gene expression of *Pkct* e *Pkca* in SOL and EDL muscles of rats at different ages. The expression of *Pkct* gradually increased with the development of EDL muscles (from neonatal age to adult age). In particular, the expression of *Pkct* increased 0.6-, 2.1-, 17-, 25-, 34-, and 49-fold in the EDL muscles of P7, P12, P33, P47, M2, and M8 rats, respectively, compared to P1 rats (**Figure 3A**, left).

*Pkct* mRNA did not change in SOL muscles in the first half of life (from P1- to P47-old rats), but thereafter gradually and significantly increased with age up to the adult stage. In particular, *Pkct* expression increased 1- and 2.38-fold in the SOL muscles of M2 and M8 rats, respectively, compared to P47 rats (**Figure 3A**), right in both SOL and EDL muscles, the mRNA of *Pkct* increased with old age. Specifically, *Pkct* in M27 rats increased by 41% and 52% in SOL and EDL muscles, respectively, compared to 8M rats.

In both EDL and SOL muscles, *Pkca* gene expression did not change in the neonatal life-stage (between P1 and P12), but increased in adolescence (P33) and did not undergo further change with increasing age until the old stage of life was reached. In particular, *Pkca* expression increased 0.6- and 1.6-fold in the SOL and EDL muscles of P33 rats, respectively, compared to P12 rats (**Figure 3B**).

### Subcellular Localization of the ClC-1 Channel in Fibers of EDL and SOL Muscles in Relation to Age

To observe the subcellular localization of ClC-1 in the fibers of EDL and SOL muscles in relation to age, we performed double-immunofluorescence staining of sections with ClC-1 and β-DG, a typical protein localized on plasma membrane (Suriyonplengsaeng et al., 2017) in transverse sections. Based on our previous results, we focused only on the P7, P12, M2, M8, and M27 age-stages. Surprisingly, the images of both the SOL and EDL muscles of P7 rats did not show an exact superposition of ClC-1 and β-DG fluorescence signals. β-DG fluorescence showed prominent plasma membrane staining, while the ClC-1 signal showed less intense staining, mostly at intracellular level.

**Figure 4:**
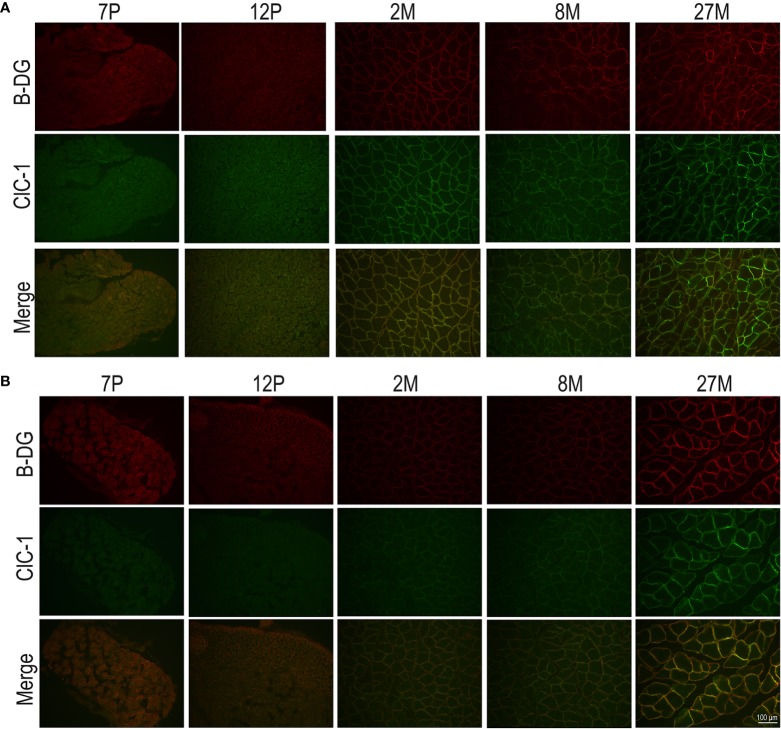
Subcellular localization of ClC-1 in EDL and SOL muscle fibers in relation to rat age obtained by epifluorescence microscopy analysis (16× magnification). The images show double-immunofluorescence with β-DG (red) and ClC-1 (green) in EDL **(A)** and SOL **(B)** fibers.

**Figure 5:**
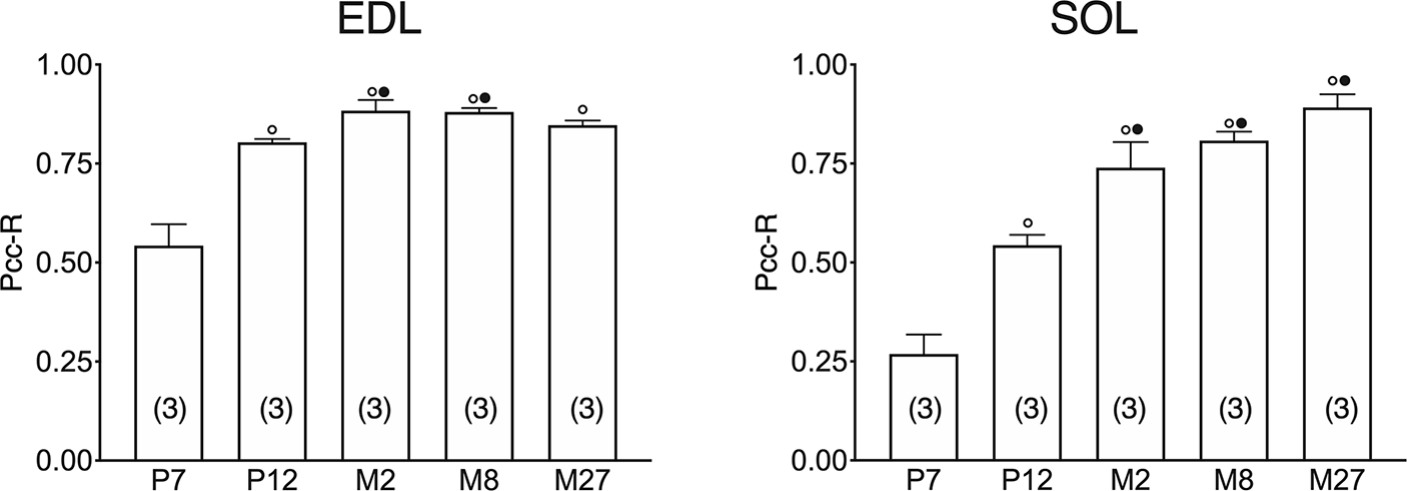
Quantification of colocalization of ClC-1 and β-DG in the fibers of EDL and SOL muscles in relation to age obtained using Pearson’s correlation coefficient and epifluorescence microscopy. The histograms show the mean ± SEM of Pcc-R obtained by comparing the distribution of ClC-1 and β-DG in three different images measured using epifluorescence microscopy (16× magnification) for each age analyzed. Significant differences between groups were evaluated using one-way ANOVA (Pcc-R in EDL muscles F=25.34, R=0.91; Pcc-R in SOL muscles F=34.72, R=0.92). Fisher’s test was used to evaluate the individual differences between groups. Significant differences (P <0.05) are expressed for ° vs. 7 Days and • vs. 12 Days.

**Figure 6:**
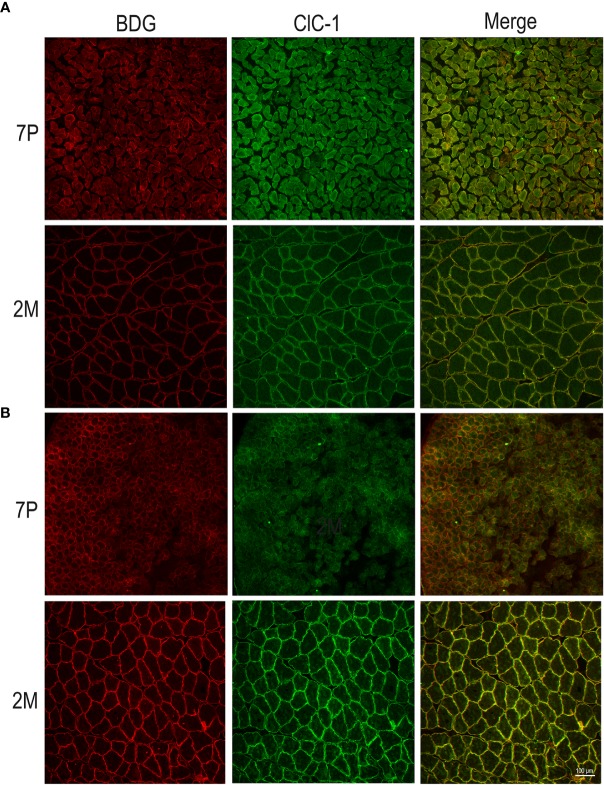
Subcellular localization of ClC-1 in EDL and SOL muscle fibers in relation to rat age using high-resolution confocal microscopy (20× magnification). Images show a transversal section of (EDL **(A)** and SOL **(B)** rat muscles 7P and 2M old obtained by double-immunofluorescence with ClC-1 (green) and β-DG (red).

**Figure 7:**
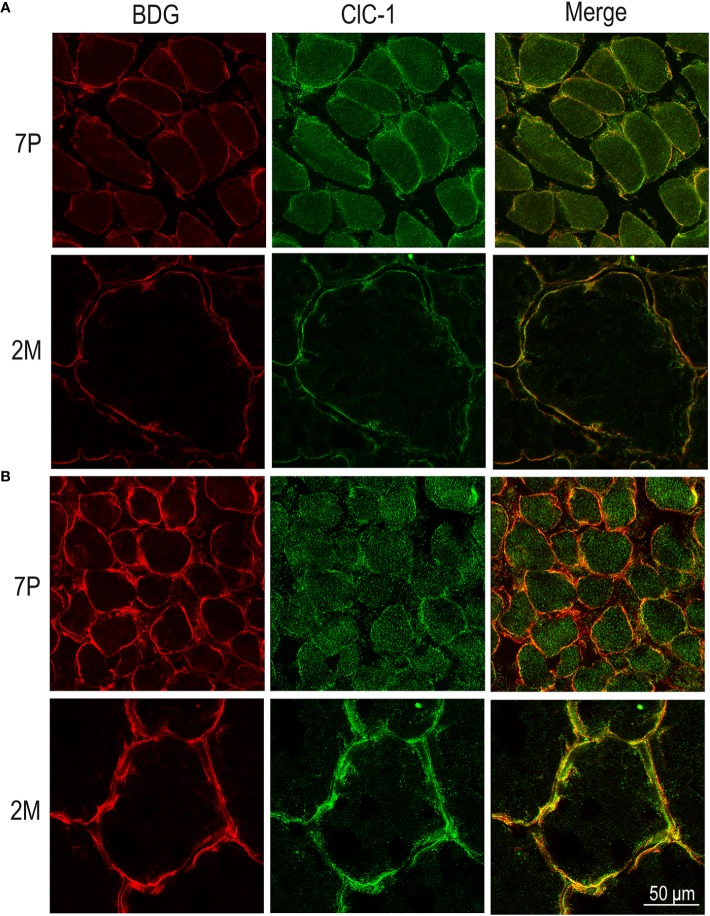
Subcellular localization of ClC-1 in EDL and SOL muscle fibers in relation to rat age using high-resolution confocal microscopy (100× magnification). Images show a transversal section of EDL **(A)** and SOL **(B)** rat muscles 7P and 2M old obtained by double-immunofluorescence with ClC-1 (green) and β-DG (red).

**Figure 8:**
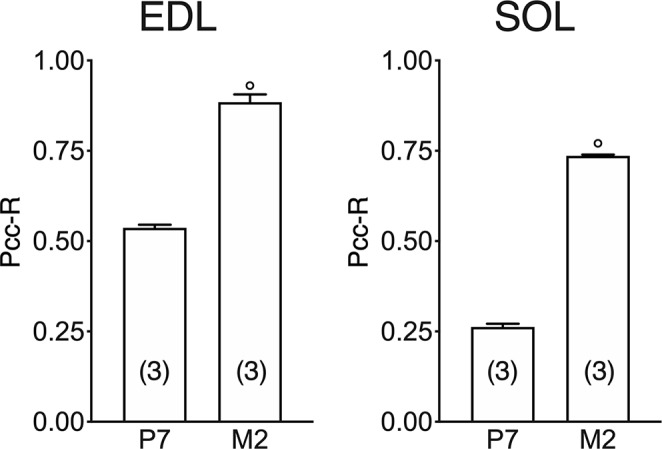
Quantification of colocalization of ClC-1 and β-DG in the fibers of EDL and SOL muscles in relation to age obtained using Pearson’s correlation coefficient and confocal microscopy. The histograms show the mean ± SEM of Pcc-R obtained by comparing the distribution of ClC-1 and β-DG in three different images measured using confocal microscopy (20× magnification) for each age analyzed. Significant differences between groups were evaluated using Student’s t-test (P <0.0001) are expressed for ° vs. 7 Days.

In both the SOL and EDL muscles of P12 rats, the signal of ClC-1 fluorescence showed double cellular localization: one part of the signal did not overlap with β-DG yielding an intracellular signal, while another part colocalized with β-DG on the plasma membrane (**Figures 4A, B**). Conversely, in muscles of M2 rats the fluorescence signal of ClC-1 strongly colocalized with β-DG on the cellular membrane and did not change with advancing age (to M8 and up to M27). To quantify the contrasting ClC-1 subcellular localization in relation to age, the previous PCC analysis was used (Babbey et al., 2006; Kanda et al., 2011) to measure ClC-1 and β-DG colocalization with epifluorescence microscopy imaging. The Pcc-R values significantly and gradually increased with age from P7 to M2 and did not undergo further change after adulthood (M8 and M27) in both the SOL and EDL muscles (**Figure 5**), suggesting that the amount of ClC-1 localized in plasma membrane increases with age from the neonatal stage until it is stabilized at the young-adult age. The values obtained by PCC analysis of confocal pictures are the following: EDL muscles of P7 rats Pcc-R=0.536 ± 0.0152 no. 3 and M2 rats Pcc-R=0.884 ± 0.037 no. 3; SOL muscles of P7 rats Pcc-R=0.262 ± 0.0153 no. 3 and M2 rats Pcc-R=0.735 ± 0.061 no. 3 (**Figures 6**–**8**).

### Correlation Between Protein and Gene CLC-1 Expression in Relation to Age

PCC analysis was performed to observe the relationship between gene and protein levels of ClC-1 during the rats’ lifespan. To improve our understanding of the role of different PKC isoforms on the posttranscriptional modulation of ClC-1 in relation to age, we performed PCC analysis between the gene expression of *Pkca* or *Pkct* and gene or protein expression of ClC-1 and mRNA in SOL and EDL muscles (**Table 3**).

**Table 3:** Pearson’s correlation coefficient analysis between protein and gene expression of ClC-1 and mRNA encoding for Pkca and Pkct in relation to age.

X data	X data	Y data	Pcc-R	P value	No. of age-groups
EDL	CLC-1 protein	*Clcn1* gene	0.871	0.011*	7
SOL	CLC-1 protein	*Clcn1* gene	0.881	0.008*	7
EDL	*Clcn1* gene	*Pkca* gene	0.806	0.028*	7
EDL	CLC-1 protein	*Pkca* gene	0.834	0.019*	7
SOL	*Clcn1* gene	*Pkca* gene	0.863	0.012*	7
SOL	CLC-1 protein	*Pkca* gene	0.638	0.129	7
EDL	*Clcn1* gene	*Pkct* gene	0.464	0.029*	7
EDL	CLC-1 protein	*Pkct* gene	0.622	0.135	7
SOL	*Clcn1* gene	*Pkct* gene	0.376	0.405	7
SOL	CLC-1 protein	*Pkct* gene	0.512	0.240	7
EDL	*Clcn1* gene	*Pkct* gene	0.650	0.161	6
EDL	CLC-1 protein	*Pkct* gene	0.907	0.012*	6
SOL	*Clcn1* gene	*Pkct* gene	0.556	0.251	6
SOL	CLC-1 protein	*Pkct* gene	0.690.	0.171	6

The table shows Pearson’s correlation coefficient value (Pcc-R), the value used to validate the hypothesis (P value) and number of groups analyzed (no. 7: P7, P12, P33, P47, M2, M8, and M27) or (no. 6: P7, P12, P33, P47, M2, and M8) between X and Y data in both SOL and EDL muscles. * indicates P value < 0.05.

A significant and very very strong correlation was observed between the ClC-1 gene and protein levels in both SOL and EDL muscles (**Table 3**). A strong correlation was also found between ClC-1 (gene or protein expression) and the values of *Pkca* mRNA in both SOL and EDL muscles. In contrast, the correlation between ClC-1 (gene or protein expression) and *Pkct* showed moderate (less or more than moderate) values under the same conditions (**Table 3**). Surprisingly, excluding the results obtained with M27 rats the correlation between ClC-1 (gene or protein expression) and *Pkct* mRNA increased, suggesting that the expression of *Pkct* and ClC-1 follows the same pattern during muscle development and the adult stage (from P7 to M8) but not during aging.

## Discussion

In accord with previous studies our data suggest that ClC-1 gene expression, detectable at birth, is modified with age. For the first time we show that the modification of ClC-1 induced by development differs according to muscle type (SOL or EDL), suggesting a strong dependency on muscular phenotype. Also, well-known difference of *Clcn1* gene expression between slow and fast phenotypes (Klocke et al., 1994; Pierno et al., 2002; Desaphy et al., 2010) is evident starting from the early weaning stage, because before this time the content of ClC-1 mRNA in the two muscle types is similar.

The different ClC-1 expression during the development of fast- and slow-twitch muscles present some functional analogies with the complex pattern of expression of different myosin heavy chain (MHCs) isoforms that characterize muscle development. Previous studies have shown that the developmental isoforms (fetal and neonatal) of MHCs are eliminated faster in muscles programmed for the fast-phenotype compared to muscles programmed for the slow-phenotype (Russell et al., 1993; Agbulut et al., 2003; Schiaffino et al., 2015). In line with these MHC trends, ClC-1 expression increases more rapidly in EDL muscles compared to SOL muscles. This difference in ClC-1 expression between the development of slow- and fast-twitch muscles can be correlated with the recent observation that ClC-1 is more expressed in type IIa than type I fibers (Thomassen et al., 2018).

An important difference observed in muscle development between the *Clcn1* gene expression profile and the fast or slow profile of MHCs is that the latter becomes definitive (as for adult muscles) at 3 months of age (Agbulut et al., 2003), while ClC-1 expression increases until 8 months of age. In this context, it is necessary to highlight that both elements can quickly change to respond to muscle physiological needs as occurs, for example, during disuse (Pierno et al., 2002; Desaphy et al., 2005; Schiaffino et al., 2015). The importance of ClC-1 in the muscles phenotype are underlined by previous results showing that the inhibition of ClC-1 in EDL can modify muscles development with a phenotype shift toward that of slow muscles (De Luca et al., 1990).

The values obtained by PCC analysis show a high correlation between gene and protein expression of ClC-1 in both fast- and slow-twitch muscle phenotypes, suggesting that there are no significant transcriptional modulations during muscle development and that most of ClC-1 mRNA is translated into protein.

To acquire more information about the possible posttranscriptional modulation of ClC-1 we analyzed the gene expression of PKC, because it is well known that ClC-1 phosphorylation induced by PKC can close the channel and reduce fiber chloride conductance (Rosenbohm et al., 1995; Rosenbohm et al., 1999; Stölting et al., 2014; Riisager et al., 2016).

We observed that the PKC isoforms gene expression is different depending the specific steps of life, suggesting that the contribution of specific PKC isoforms is different in skeletal muscles development and aging. In particular, *Pkct* gene expression gradually increases throughout the life course, highlighting the important role of this isoform in all stages of muscle development and a different role in muscular adaptation to aging. This hypothesis is in line with previous observations reporting that mice deficient in PKC show significant alterations in embryonic development (Carracedo et al., 2014). Furthermore, it was demonstrated that PKC promotes the differentiation of myotube cells and muscle regeneration through the regulation of genes correlated with fiber growth, such as *MyoD* and *MyoG* (Di Marcantonio et al., 2015). On the other hand, *Pkca* gene expression increases only during the transition from the weaning age to young-adult age in both muscle types analyzed. This observation suggests that the role of *Pkca* in muscle growth is crucial in this particular stage of life. Further studies are warranted to shed light on the increase of *Pkca* only in one specific stage of muscle development.

Supposing that at a specific age the more expressed isoform of PKC has greater potential to modulate the ClC-1 channel compared to the less expressed isoform, then it is possible to hypothesize that the different expressions of *Pkc* isoforms in relation to age can infuence the posttranscriptional modulation of ClC-1. Our study showed that the PCC correlation between *Clcn1* and *Pkct* is high when the data related to old age were excluded from the analysis. This observation suggests that *Pkct* is particularly important for ClC-1 posttranscriptional modulation during muscle development. On the contrary, aging simultaneously induces a decrease of ClC-1 gene or protein expression and an increase of *Pkct* mRNA. This evidence is in line with previous data that show a reduction of gCl induced by aging (De Luca et al., 1994).

The trend of *Pkca* gene expression seems to suggest that after the young-adult stage a more precise modulation of the ClC-1 channel is necessary with the contribution of both *Pkca* and *Pkct* to optimally stabilize muscle function. On this basis, our results suggest that the modulation of ClC-1 mediated by PKC depends on the different isoforms that intervene in different ways and at different age-stages.

The different trend of *Clcn1* and *Pkc* expression during development in slow and fast muscles can be important to support the correct muscle growth. Our results suggest that during development fast muscle fibers need faster increase of ClC-1 expression with respect to slow fibers. This observation is in line with the well-known evidence that the adult fast muscles express more ClC-1 with respect to slow one for functional needs (Klocke et al., 1994; Pierno et al., 2002; Desaphy et al., 2010). Indeed, in the fast muscles the ClC-1 channel is responsible for the high chloride conductance (gCl), that functions to stabilize the resting membrane potential and to control sarcolemma excitability. In fact, a dramatic decrease of gCl due to mutation of ClC-1 gene is responsible for abnormal hyperexcitability typical of hereditary myotonia (Pierno et al., 1999; Desaphy et al., 2013; Stölting et al., 2014). In contrast, in the postural soleus muscle the lower gCl is important to sustain its antigravity function (Pierno et al., 2002). Indeed, the slow-twitch muscle is characterized by a typically low gCl that contributes to maintain appropriate sarcolemma excitability. This allows the slow fibers to be tonically active and resistant to fatigue.

As already described, the PKC is important to regulate ClC-1 activity (Rosenbohm et al., 1995; Fahlke et al., 1996; Pedersen et al., 2009). Since chloride current counteracts action potential rise, the PKC activation change the voltage-dependent open probability by phosphorylating and closing the channel (Hsiao et al., 2010). This is important to starts muscle excitability (Rosenbohm et al., 1995; Fahlke et al., 1996; Pedersen et al., 2009; Hsiao et al., 2010; Riisager et al., 2016). In our experiments the PKC starts to be significantly expressed after P12 in the fast-twitch EDL, a muscle used in powerful bursts of movements. In Sol muscle the PKC is more active and then responsible for ClC-1 inhibition of activity. In previous studies we demonstrated higher activity of PKC in soleus muscle, indeed the application of chelerythrine, an inhibitor of PKC, increased the gCl toward that of EDL muscle (Pierno et al., 2007).

It is also known that PKC plays an important role in neuromuscular junction (NMJ) regulation during the first 2–3 weeks postnatal period. It is also important in the modulation of genes correlated with cells differentiation or muscle regeneration, suggesting additional roles in muscle phenotype maturation (Dobrowolny et al., 2018).

Surprisingly, but similar to gene and protein expression, the subcellular localization of ClC-1 is not definitive at birth. Changes in ClC-1 subcellular localization are evident only in the early stages of muscle development, but become definitive very quickly before the weaning stage. This evidence suggests that in the first phases of muscle development ClC-1 is localized in the intracellular pool and different cell stimuli are needed to drive translocation to the plasma membrane. This hypothesis is in agreement with previous evidence suggesting that ClC-1 can be differently localized according to different cellular stimuli (Papponen et al., 2005; Pedersen et al., 2016).

It has been highlighted that during the embryonic and postnatal age a particular alternative splicing variant of *Clcn1*, containing exon 7a, plays a role in muscle development (Wischmeyer et al., 1993; Cooper, 2007). In healthy mice, gene expression of the 7a exon-alternative splicing variant of *Clcn1* decreases with age while the mRNA of the traditional *Clcn1* isoform increases (Lueck et al., 2007).

Differently, the information about the same splicing variant of *Clcn1* (correlated with exon 7), in the rat are few. It is known that the exon 7 of *Clcn1* is responsable of three splicing variants in the rat (Zhang et al., 2004). Surprisingly, these splicing variants of *Clcn1* have been observed in the astrocytic glial cells but not in the skeletal muscles of adult rats (Zhang et al., 2004), although they are not functional, since did not show any electrophysiological activity in *Xenopus Oocytes* (Zhang et al., 2004).

Thus, we cannot exclude that the intracellular signal of ClC-1 is correlated with particular ClC-1 alternative splicing which is specific to the fetal-neonatal age, because today antibodies capable of discriminating full-length isoforms for fetal or neonatal isoforms are not available.

It cannot be excluded that the intracellular ClC-1 signal may depend on the numerous changes taking place during the trasformation of the fibers into the mature architecture of adult muscles as occurs, for example, in the biogenesis and maturation of t-tubule structure. We emphasize that t-tubule biogenesis and the intracellular ClC-1 signal do not take place in the same stages of muscle development or at the same age. Specifically, the first defined t-tubules can be observed at 14-16 days of embryonic life, and final maturation is complete after 3 weeks (Al-Qusairi and Laporte, 2011). On the other hand, the intracellular ClC-1 signal is observable at 7-12 days after birth and, therefore, does not perfectly correspond with the time plan of t-tubule biogenesis and maturation.

Further studies are needed to clarify the physiological role and regulation of the intracellular pool of ClC-1 observed in the early stages of muscle development.

The hypothesis that the ClC-1 channel translocates from an intracellular pool to the plasma membrane during the first stages of muscle development represents an interesting focus for future pharmacological strategies. Indeed, it is known that only poorly selective blockers and a few positive modulators exist for ClC-1. For this reason, the drugs prescribed for treating myotonia include the anti-arrhythmic agent mexiletine and the anti-epileptic agent lamotrigine (Andersen et al., 2017; Stunnenberg et al., 2018) to induce a reduction of muscle tone and suppress action potential firing in skeletal muscles. It has also been observed that the application *in vivo* or *in vitro* of growth hormone, insulin-like growth factor-1, taurine and angiotensin II can stimulate intracellular biochemical pathways and increase gCl in skeletal muscles (De Luca et al., 1998; Desaphy et al., 1998; Pierno et al., 1998; Cozzoli et al., 2014; Pierno et al., 2014).

The mutations of ClC-1 associated with myotonia congenita can be correlated to anomalous gating-permeation or reduced ClC-1 protein abundance at the plasma membrane (Lee et al., 2013; Jeng et al., 2020). This distinction, together with the new and important advance in knowledge of the mechanisms behind proteostasis (Peng et al., 2016), biogenesis (Chen et al., 2015) and those governing trafficking to the surface membrane (Peng et al., 2018), has opened the doors to the novel pharmacological approach for increasing ClC-1 in plasma membrane (Altamura et al., 2018; Jeng et al., 2020). By studying the biophysical mechanism to induce an increase of ClC-1 in plasma membrane, certain inhibitors of ligase E3 (Lee et al., 2013) or blockers of ubiquitin inhibitors (Chen et al., 2015) are emerging. The hypothesis that the ClC-1 channel translocates from an intracellular pool to the plasma membrane during the first stages of muscle development represents an attractive starting point for innovative pharmacological strategies aimed at identifying novel drugs able to modulate defective membrane trafficking.

## Data Availability Statement

The datasets generated for this study are available on request to the corresponding author.

## Ethics Statement

The animal study was reviewed and approved by General Direction of animal health care and veterinary drugs of the Italian Ministero della Salute (Italy).

## Author Contributions

GC designed the studies. EC designed and conducted the Western blot experiments. AF and VB performed the qPCR experiments. AF and AC conducted the histological experiments. AF conducted the PCC analysis. GN supervised and interpreted the histological results. GC and EC analyzed results and organized the figures and statistical analyses. GC drafted the manuscript. GC, SP, and PI contributed to the interpretation of the data. GC and SP critically revised the manuscript.

## Conflict of Interest

The authors declare that the research was conducted in the absence of any commercial or financial relationships that could be construed as a potential conflict of interest.

## Abbrevations

ClC-1, chloride channel 1; PKC, protein kinase C; SOL, soleus; EDL, extensor digitorum longus; *Clcn1*, chloride channel 1 gene; gCl, plasma membrane Cl conductance; GLUT4, glucose transporter type 4; P, postnatal-days; M, postnatal-months; qPCR, quantitative polymerase chain reaction; *Actb*, beta-actin gene; *Gapdh*, glyceraldehyde-3-phosphate dehydrogenase gene; *Hprt1*, phosphoribosyltransferase 1 gene; *Pkct*, protein kinase C theta gene; *Pkca*, protein kinase C alpha gene; PCC, Pearson’s correlation coefficient analysis; β-DG, beta-dystroglycan; Pcc-R, Pearson's correlation coefficient values; *MyoD*, myogenic differentiation gene; *MyoG*, myogenic factor gene; AQP-1, Aquaporin-1.
