# Supplementary material for: Changes in Expression and Cellular Localization of Rat Skeletal Muscle ClC-1 Chloride Channel in Relation to Age, Myofiber Phenotype and PKC Modulation
Source: Front Pharmacol. 2020 May 15;11:714. doi: 10.3389/fphar.2020.00714 (PMC7243361; doi:10.3389/fphar.2020.00714)
Supplement: Supplementary file 1 [file DataSheet_1.pdf]

Supplementary Material:

Changes in expression and cellular localization of rat skeletal muscle ClC-1 chloride channel in relation to age, myofiber phenotype and PKC modulation

Antibody Validation

Validation experiments were performed to acquire additional information on antibody specificity for the application of immunofluorescence and Western blot analyses. In particular, four types of Western blot validation experiments were performed. In the first group we used the Hek cell line which spontaneously does not express ClC-1 transfected with empty plasmid or with ClC-1 plasmid. The Western blot analysis, conducted for the entire electrophoretic run, shows a single band only in Hek-ClC-1 cells with a molecular weight between 100and 150 kDa corresponding to 110-120kDa expected for the ClC-1 protein (Fig. S1).

To acquire knowledge on the specificity of ClC-1 antibody in a more complex biological system of cells lines, in the second group of experiments we performed immunoblotting with ClC-1 antibody on the entire electrophoretic run in soleus (SOL) and extensor digitorum longus (EDL) muscles of rats 8 months of age. Furthermore, to detect all bands in the samples we extended the time of revelation on the Chemidoc imaging system. In both analyzed muscle types, results show a band with a molecular weight between 100 and 150 kDa (deductible from the protein marker) possibly corresponding to 110-120kDa expected for ClC-1 (Fig. S2). Fig. S2 also shows a band corresponding to 75 kDa of the protein marker, which cannot correspond to any isoform of ClC-1 probably due to a polyclonal-type antibody. Indeed, the five isoforms of ClC-1 observed in the astrocytic glial cells have a different molecular weight (Zhang et al. 2004).

In the third group of experiments we analyzed ClC-1 protein expression in different types of rat tissue, because it is known that ClC-1 is almost exclusively expressed in skeletal muscle, as previously demonstrated by mRNA analysis (Steinmeyer, 1991). In line with previous studies ClC-1 is detectable only in skeletal muscles, in particular EDL muscles, and is not detectable in any other analyzed tissue white and brown fat, heart, liver, uterus, testis and lung (Fig. S3). In the in the fourth group of experiments we verified the linearity of the ClC-1 immunoblot quantification system by using the Hek cells transfected with the ClC-1 vector. Immunoblotting with ClC-1 antibody was

performed using different quantities of total protein extract obtained from cells (Fig. S4 line A and S4 line B). A linear correlation analysis between the micrograms of total protein and the volume intensity of bands obtained by Western blotting was found (Fig. S4 box C).

To obtain more information on antibody specificity for the application of immunofluorescence we performed two types of validation experiments. In the first experimental group we conducted immunofluorescence with ClC-1 antibody in Hek cells transfected with empty vector or with the ClC-1 vector. Fig. S5 shows fluorescence of the ClC-1 protein in Hek-ClC-1 cells and a background fluorescence noise in the Hek cells.

In the second group of experiments the SOL muscles of P12 rats were used to observe the green-fluorescence signals under different conditions compared to the beta-dystroglycan (β-DG) red-fluorescence signals. The same experiment was performed to analyze ClC-1 localization in relation to age (Fig. S6 line A). We compared the fluorescence signals of the previous images with the fluorescence signals of a protein not expressed in skeletal muscles to acquire nonspecific signals caused by the absence of bound with primary antibody (Fig. S6 line B). Aquaporin-1 (AQP-1) was used as an unexpressed protein in skeletal muscle fiber, because it is known that AQP-1 is expressed on the endothelial cells of capillaries and not in the plasma membrane of muscle fibers (Frigeri et al. 2004). To obtain the signal caused by background noise, which is specific of green-secondary antibody, we performed an immunofluorescence stain only with β-DG primary antibody (red) (Fig. S6 line C). The merged image of line A shows that a fraction of the ClC-1 signal is co-localized to the signal of β-DG. The merged images of lines B and C depict observed green non-specific signals (inside and outside the fibers) in contrast with the β-DG signals on the plasma membrane (red).

Methods:

HEK293 cells and preparation of Western blot and immunofluorescence analyses

Human embryonic kidney (HEK293) cells were transiently transfected with empty plasmid (Hek) or with plasmid containing full-length hClC-1 cDNA (Hek-ClC-1) by using the calcium phosphate precipitation method. After 48 h, the cells were subject to the Western blot or immunofluorescence experiment. For Western blotting the cells were harvested in 200 ml of cold RIPA buffer (150 mM NaCl, 20 mM Tris-HCl pH=7.45, 5 mM EDTA, 1.5% NP-4, 1 mM Na3VO3, 10 mg/ml PMSF, and a protease inhibitor cocktail) and incubated for 10 min on ice. To complete cellular lysis, cell suspensions were passed through a syringe with a needle 10-15 times. After 20 min of incubation on ice, the cell lysates were centrifuged at 14,000 rpm for 30 min at 4°C, and the supernatant was

collected. Cell proteins (5 μg) were separated on SDS-PAGE and the other Western blot phases were carried out as described in the main text. The cells used for immunofluorescence experiments were deposited on glass coverslips. On the day of the experiments they were fixed and permeabilized with 100% ice-cold methanol for 5 min to continue with immunofluorescence analysis as described in the main text.

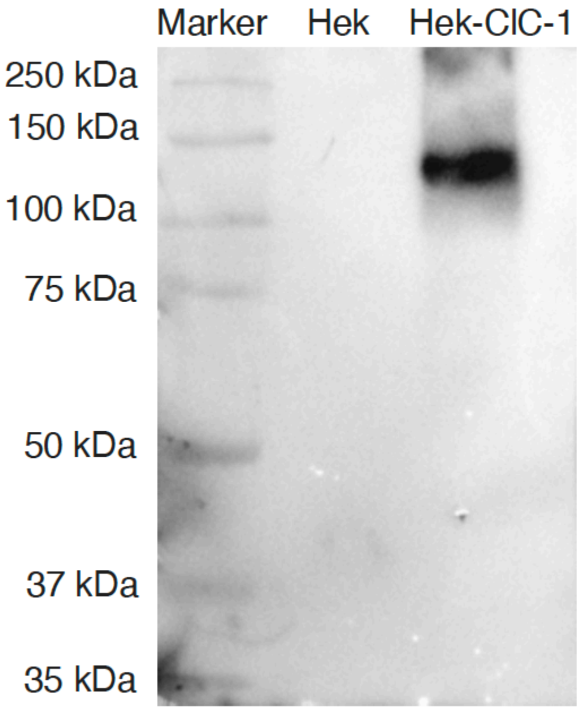

Figure S1. Western blot of ClC-1 protein in Hek cells transfected with empty plasmid (Hek) and with ClC-1 plasmid (Hek-ClC-1) compared to protein marker for electrophoresis.

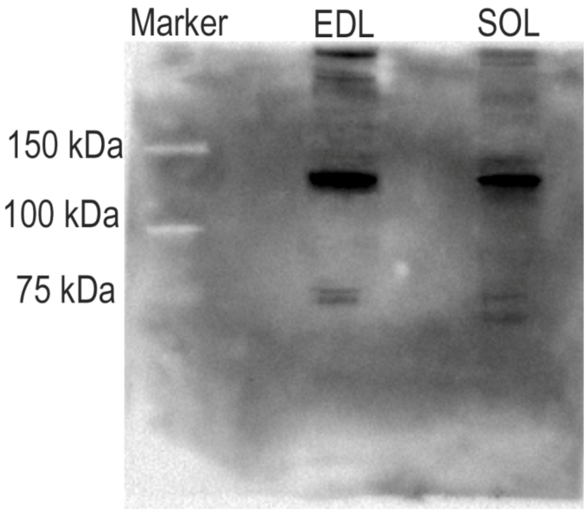

Figure S2. Western blot of ClC-1 protein in extensor digitorum longus (EDL) and soleus (SOL) muscles of 8-month-old rats compared with protein marker for electrophoresis.

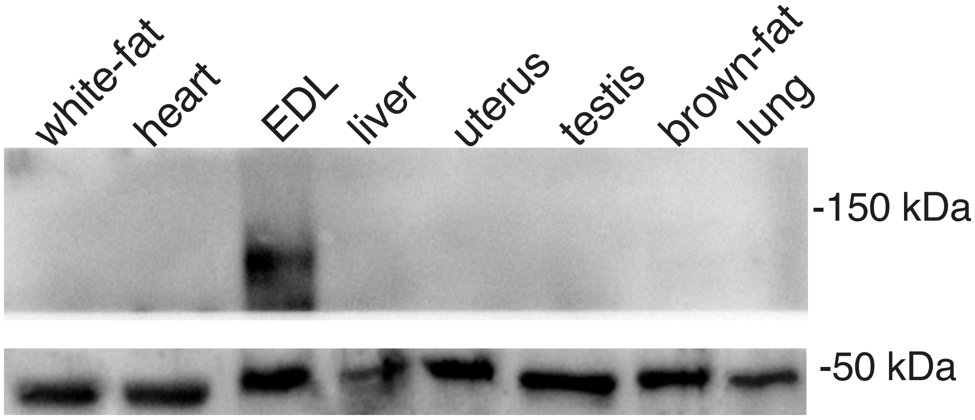

Figure S3. Tissue distribution of ClC-1 protein. The image shows the protein expression of ClC-1 in different types of rat tissue – white and brown fat, heart, extensor digitorum longus (EDL), liver, uterus, testis, and lung – compared with β-actin expression

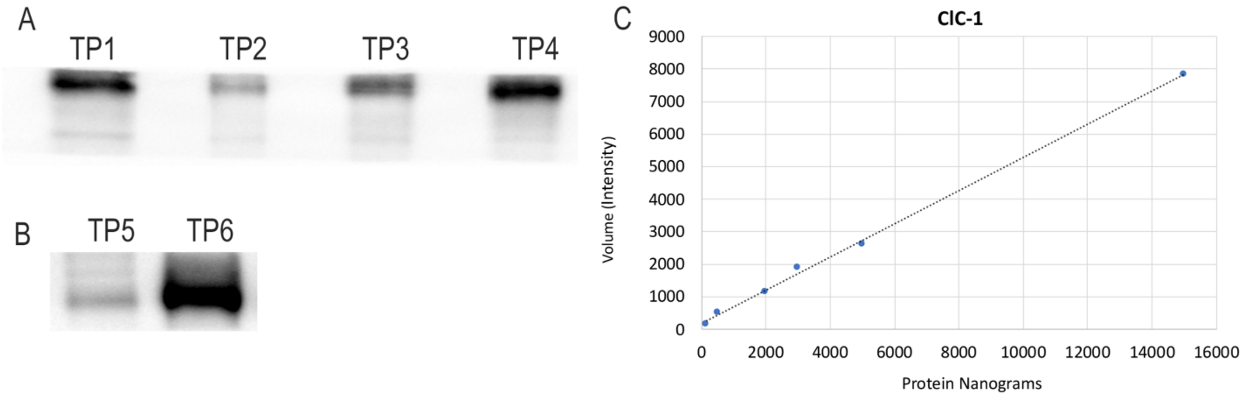

Figure S4. Western blot of ClC-1 protein in Hek cells transfected with ClC-1 vector (Hek-ClC-1) performed using different quantities of total protein extract (TP). Western blot figure A point TP1: 5 μg total protein; point TP2: 0.5 μg total protein; point TP3: 2 μg total protein; point TP4: 3 μg total protein. Western blot figure B point TP5: 0.15 μg total protein and point TP6:15 μg total protein. Figure C: linear regression graph obtained comparing the micrograms of total protein with the volume intensity of bands obtained by the two Western blotting.

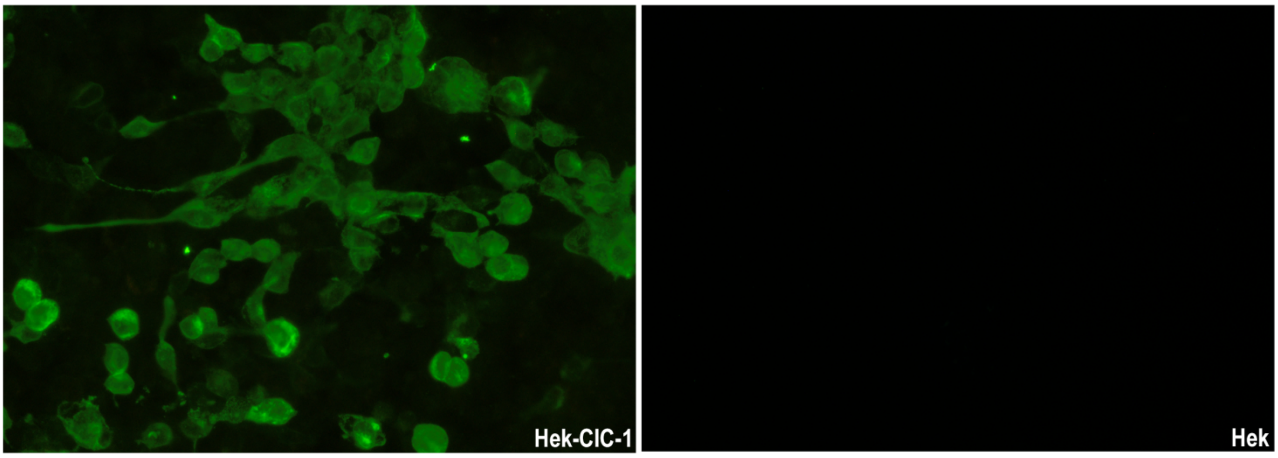

Figure S5. Immunofluorescence with ClC-1 of Hek transfected with ClC-1 vector (Hek-ClC-1) and with empty vector (Hek).

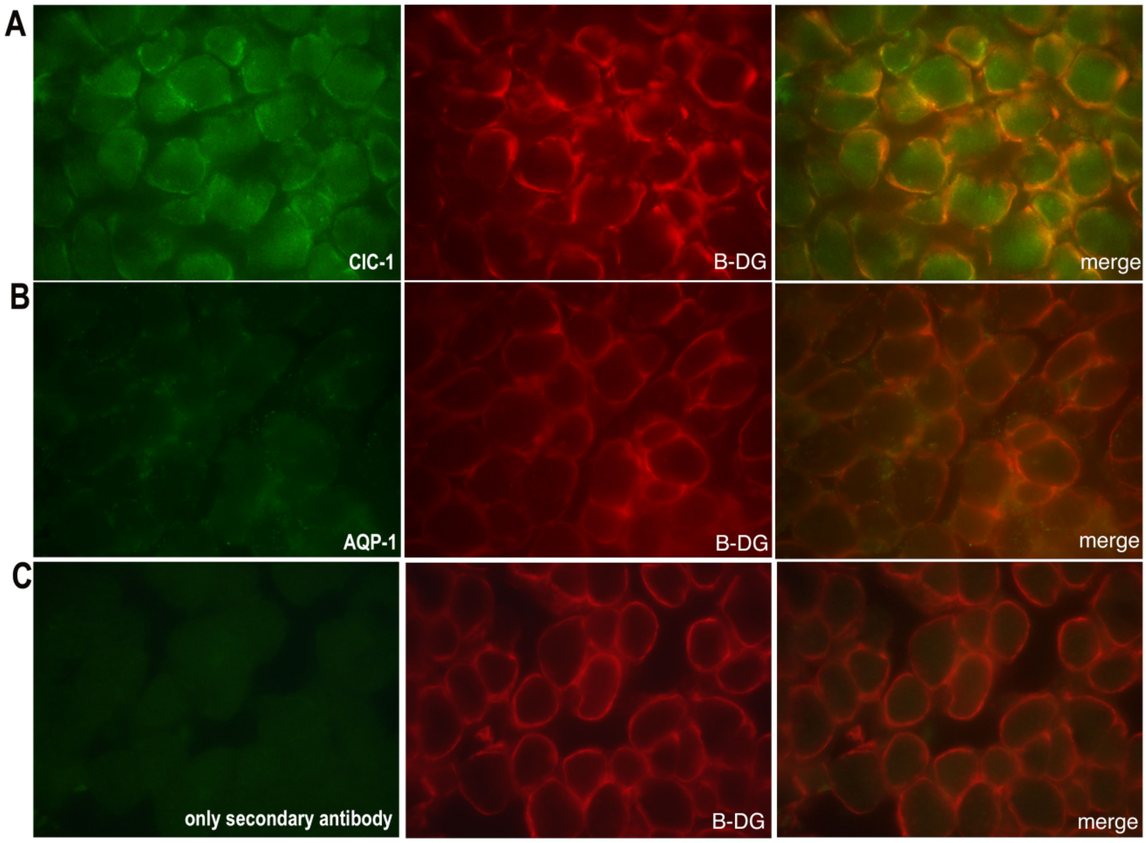

Figure S6. Double-immunofluorescence analysis on soleus (SOL) muscles of P12 rats. The signals of ClC-1 (A), AQP-1 (B) and background noise of secondary antibody (C) are shown in green. The β-DG signal in all cell lines is shown in red.
